# Antimalarial Drug Quality in the Most Severely Malarious Parts of Africa – A Six Country Study

**DOI:** 10.1371/journal.pone.0002132

**Published:** 2008-05-07

**Authors:** Roger Bate, Philip Coticelli, Richard Tren, Amir Attaran

**Affiliations:** 1 Africa Fighting Malaria, Washington, D. C., United States of America; 2 American Enterprise Institute, Washington, D. C., United States of America; 3 Institute of Population Health & Faculties of Law and Medicine, University of Ottawa, Ottawa, Canada; University of Montreal, Canada

## Abstract

A range of antimalarial drugs were procured from private pharmacies in urban and peri-urban areas in the major cities of six African countries, situated in the part of that continent and the world that is most highly endemic for malaria. Semi-quantitative thin-layer chromatography (TLC) and dissolution testing were used to measure active pharmaceutical ingredient content against internationally acceptable standards. 35% of all samples tested failed either or both tests, and were substandard. Further, 33% of treatments collected were artemisinin monotherapies, most of which (78%) were manufactured in disobservance of an appeal by the World Health Organisation (WHO) to withdraw these clinically inappropriate medicines from the market. The high persistence of substandard drugs and clinically inappropriate artemisinin monotherapies in the private sector risks patient safety and, through drug resistance, places the future of malaria treatment at risk globally.

## Introduction

Malaria surged in sub-Saharan Africa in the 1990s, due in part to increased resistance to chloroquine and sulfadoxine-pyrimethamine (SP).[1] Exposure to substandard antimalarial drugs likely exacerbated this trend.[2], [3], [4] Pressure from malaria scientists prompted wholesale adoption of artemisinin-based combination therapies (ACTs) by endemic country governments and donors.[5] In January 2006, the WHO issued new treatment guidelines for the first time in 20 years recommending ACTs for the treatment of uncomplicated malaria. The WHO further called to end the production and marketing of artemisinin monotherapies in order to protect these formulations against parasitic resistance. According to the WHO's antimalarial drug quality database, every national treatment policy in sub-Saharan Africa except Swaziland and Cape Verde now recommends treating uncomplicated malaria with ACTs.

These well-intentioned changes might remain unrealized and vulnerable. Substandard ACTs, and “legitimate” artemisinin monotherapies, are unparalleled threats driving clinical failure of malaria treatment.[6], [7], [8] Especially in Sub-Saharan Africa, governments lack the ability through customs and policing to stop these medicines entering the private market, where many (oftentimes most) persons buy their treatment.[9], [10] Unless these medicines are of a consistently high quality, the great strides made in recent years to transition from chloroquine and SP to ACTs could be imperiled by drug resistance. This study is the first to sample the quality of medicines throughout the geographic band of hyper- and holoendemic *P. falciparum* malaria which stretches unbroken from West, to Central, to East Africa—the world's worst.[11]

## Results

195 treatment packs were tested, producing 210 sample results. The difference between tests and results is explained in that co-packaged, but not co-formulated ACTs, such as artesunate and amodiaquine were tested as individual monotherapies.

Overall 35% (73/210) of tested samples were substandard and failed either TLC or dissolution tests (See Table 1). Of the specific pharmaceutical types, failure by TLC, dissolution or both, occurred in 38% of SP, 48% of amodiaquine, 24% of mefloquine, 31% of artesunate, 27% of artemether, 55% of dihydroartemisinin and 19% of artemether-lumefantrine fixed-dose combinations.

**Table 1:** Testing results by formulation and country purchased for TLC and dissolution^i^,^ii^

	Ghana	Kenya	Nigeria	Rwanda	Tanzania	Uganda	Total
Sulfadoxine-pyrimethamine	50% (3/6)	38% (6/16)	50% (1/2)	50% (3/6)	27% (3/11)	33% (3/9)	**38% (19/50)**
Amodiaquine	33% (2/6)	50% (4/8)	25% (1/4)	-	100% (2/2)	56% (5/9)	**48% (14/29)**
Mefloquine	0% (0/1)	-	50% (1/2)	-	0% (0/3)	27% (3/11)	**24% (4/17)**
Artesunate	38% (3/8)	0% (0/4)	33% (2/6)	-	31 (4/13)	33% (6/18)	**31% (15/49)**
Artemether	0% (0/3)	100% (1/1)	-	-	-	29% (2/7)	**27% (3/11)**
Dihydro-artemisinin	40% (2/5)	56% (5/9)	100% (1/1)	-	50% (2/4)	67% (2/3)	**55% (12/22)**
Artemether-lumefantrine fixed-dose combination	38% (3/8)	0% (0/4)	14% (1/7)	0% (0/3)	0% (0/1)	22% (2/9)	**19% (6/32)**
Total	**35% (13/37)**	**38% (16/42)**	**32% (7/22)**	**33% (3/9)**	**32% (11/34)**	**35% (23/66)**	**35% (73/210)**

i. Percentages are supported by (total that failed either dissolution or TLC/total treatments tested)

ii. Co-packaged ACTs are listed as individual monotherapies

Artemisinin monotherapy, which the WHO rejects as inherently substandard treatment even when its dosage is correct, remains common in Africa. 33% (64/195) of all treatment packs tested were artemisinin monotherapies, and 42% (27/64) failed either TLC or dissolution tests. 78% (50/64) were manufactured after the WHO's January 2006 appeal to halt monotherapy production. A further sign that certain manufacturers of artemisinin monotherapies do not take clinical efficacy seriously was the tremendous heterogeneity in expiry dates: 10 listed an expiration date of two years, while most (42/64) listed three years. Five listed an expiration date of four years, and seven lacked either a manufacture date or both a manufacture date and expiration date. Names of the offending artemisinin monotherapy producers in this study were forwarded to the WHO.

The authors did not attempt a forensic examination of trademarks or product designs to differentiate between products that were merely substandard and those which were deliberately counterfeited, as neither is clinically suitable. However, there was an apparent trend in that failing products more often originated or were claimed to originate from poorer parts of the world with weaker regulatory systems: failure rates were 48% (30/63) for Africa, 32% (29/90) for Asia and 24% (12/50) for Europe (See Table 2). Only four US-manufactured samples were tested; none failed.

**Table 2:** Testing results by region of manufactureiii

Region of manufacture	Total Samples Failing TLC or Dissolution	Total Samples Tested	Percent Failed
Africa	30	63	**48%**
Asia	29	90	**32%**
Europe	12	50	**24%**
US	0	4	**0%**

iii. Manufacturer information not available for 3 tested sampless

## Discussion

This study sheds light on the availability and relative quality of private sector antimalarials in Africa's private sector. In countries situated in the world's most intense region of holoendemic and hyperendemic *P. falciparum* malaria, where the difference between a proper and a bogus medicine cannot be surpassed, various substandard therapies and clinically inappropriate monotherapies remain widely available, with between a quarter and over half of products sold in urban and peri-urban pharmacies failing basic quality testing. We do not quantitatively estimate the public health impact of this crisis, but it must be staggering.

The WHO has taken diplomatic steps to fight this problem. It coordinates a passive reporting system for substandard medicines, for example, and World Health Assembly Resolution WHA60.18 of May 2007 committed member states to cease production and marketing of artemisinin monotherapies. Our study results suggest these diplomatic efforts alone are not making a sufficient impact in the field. To be effective, the WHO needs its partners to support policy change, which we describe here.

Other international agencies must apply leverage, apart from the WHO's sole diplomatic efforts. Major financiers of malaria treatment, such as the Global Fund and the World Bank, should make aid conditional on countries de-listing oral artemisinin monotherapies from national formularies. The World Trade Organisation, which sets the rules of global commerce, should enact rules prohibiting the international trade in artemisinin monotherapies and reducing the tariffs on proper medicines to zero. These steps will ensure that less money flows to the inappropriate monotherapies, and that ACTs are made less expensive. The political traction to accomplish these steps should come from the Group of Eight leading industrial countries, who declared ACTs a global priority in 2005.

Effort must also be made to reverse the various initiatives which now spend public money on inadequately regulated medicines. The Global Fund, for example, finances the purchase of medicines whose quality is not approved by the WHO's prequalification scheme or any developed country's regulatory authority–known as “Option C” medicines. According to an internal report published by the Wall Street Journal, the World Bank recently discovered in an audit that malaria medicines it purchased in India from a local manufacturer were clinically substandard.

These incidents argue strongly for a rule against purchasing locally-manufactured medicines, except where those medicines have received regulatory approval from a developed country or the WHO's prequalification scheme. The WHO's current practice, which regards a medicine as good enough if it has *applied* for prequalification, but not necessarily passed the WHO quality standards and *received* prequalification, is an unprincipled distortion of the rules. Similarly stringent rules must inure to the proposed $1.9 billion Affordable Medicines Facility for malaria, which intends to provide clinically effective ACTs at retail outlets where they are needed. Unless this initiative employs standards higher than the current Global Fund and World Bank ones and strengthens post-market surveillance, it risks to expand the supply of the substandard and inappropriate treatments found in this study, which would be disastrous in clinical and drug resistance terms both.[13]

Further, this study demonstrates that at the local level, the capacity for basic drug quality tests to strengthen post-market surveillance can be deployed with relative ease. A Minilab® or equivalent technology costs about $4,000, requires modest training, and can be run in an ordinary air-conditioned room. The marginal cost to test 10 samples out of 10,000 (for a sampling ratio of 0.1%) is about $2, and can be completed in under an hour. Training programs for district level malaria officers and regulatory officials in Southeast Asia could be replicated in sub-Saharan Africa.[14] Such simple and cheap technology should be distributed more widely, to the ministry of health, police, customs services and non-governmental organizations. A decentralized effort of this kind, with drug quality watchdogs at several layers of government and in civil society, could be effectively tried in a country for only tens of thousands of dollars.

## Materials and Methods

A simple sampling protocol was developed in line with similar studies.[12] Antimalarial drugs were obtained by local nationals from randomly selected private pharmacies in the major cities of six African countries within the high endemicity band. Study agents posed as customers were asked to purchase a sample lot of antimalarial tablet formulations available, namely: SP, amodiaquine, mefloquine, artemisinin monotherapies and any ACTs. Agents were instructed not to purchase chloroquine. Treatment packs were maintained either in the manufacturer's original packaging or loose, and stored at ambient temperature until testing. Tests were completed within 40 days of sample collection.

The Global Pharma Health Fund e.V. Minilab® was used to run semi-quantitative thin-layer chromatography (TLC) and dissolution tests on each sample to determine the presence and relative concentration of active ingredients. Each test was run in duplicate, with the generous assumption that the result more consistent with the reference was recorded. The Minilab® protocols award products a “pass” if they have 80% or more of the labeled active ingredient(s) (note there is no upper-bound limit). For fixed-dose combinations and SP, “pass” was awarded only if both active ingredients met this standard.

## References

[1] Gomes M, Wayling S, Pang L (1998). Interventions to improve the use of antimalarials in south-east Asia: an overview.. Bull World Health Organ.

[2] Minzi OMS, Moshi MJ, Hipolite D, Massele AY, Tomson G (2003). Evaluation of the quality of amodiaquine and sulphadoxine/pyrimethamine tablets sold by private wholesale pharmacies in Dar Es Salaam Tanzania.. Journal of Clinical Pharmacy and Therapeutics.

[3] Amin AA, Snow RW, Kokwaro GO (2005). The quality of sulphadoxine-pyrimethamine and amodiaquine products in the Kenyan retail sector.. Journal of Clinical Pharmacy and Therapeutics.

[4] Basco LK (2004). Molecular epidemiology of malaria in Cameroon. XIX. Quality of antimalarial drugs used for self-medication.. Am J Trop Med Hyg.

[5] Attaran A, Barnes KI, Curtis C, D'Alessandro U, Fanello CI (2004). WHO, the Global Fund and medical malpractice in malaria treatment.. The Lancet.

[6] Dondorp AM, Newton PN, Mayxay M, Van Damme W, Smithuis FM (2004). Fake antimalarials in Southeast Asia are a major impediment to malaria control: multinational cross-sectional survey on the prevalence of fake antimalarials.. Trop Med Int Health.

[7] Hall KA, Newton PN, Green MD, De Veij M, Vandenabeele P (2006). Characterization of counterfeit artesunate antimalarial tablets from southeast Asia.. Am J Trop Med Hyg.

[8] Newton PN, McGready R, Fernandez F, Green MD, Sunjio M (2006). Manslaughter by Fake Artesunate in Asia—Will Africa Be Next?. PLoS Med.

[9] Snow RW, Peshu N, Forster D, Mwenesi H, Marsh K (1992). The role of shops in the treatment and prevention of childhood malaria on the coast of Kenya.. Trans R Soc Trop Med Hyg.

[10] Goodman C, Brieger B, Unwin A, Mills A, Meek S (2007). Medicine Sellers and Malaria Treatment in Sub-Saharan Africa: What Do They Do and How Can Their Practice Be Improved?. Am J Trop Med Hyg.

[11] Snow RW, Guerra CA, Noor AM, Myint HY, Hay SI (2005). The global distribution of clinical episodes of Plasmodium falciparum malaria.. Nature.

[12] Lon CT, Tsuyuoka R, Phanouvong S, Nivanna N, Socheat D (2006). Counterfeit and substandard antimalarial drugs in Cambodia.. Transactions of the Royal Society of Tropical Medicine and Hygiene.

[13] Green MD (2006). Antimalarial drug resistance and the importance of drug quality monitoring.. J Postgrad Med.

[14] Vijaykadga S, Cholpol S, Sitthimongkol S, Pawaphutanan A, Pinyoratanachot A (2006). Strengthening of national capacity in implementation of antimalarial drug quality assurance in Thailand.. Southeast Asian J Trop Med Public Health.

